# Residual calcified material volume of β–tricalcium phosphate with platelet-rich fibrin in unilateral alveolar bone graft

**DOI:** 10.1186/s40902-024-00420-1

**Published:** 2024-03-01

**Authors:** Chon T. Ho Nguyen, Minh H. Bui, Phuong H. Lam

**Affiliations:** 1 Department of Maxillofacial Surgery, Faculty of Odonto-Stomatology, University of Medicine and Pharmacy at Ho Chi Minh City, Ho Chi Minh City, 700000 Vietnam; 2 Department of Maxillofacial Surgery, National Hospital of Odonto-Stomatology Ho Chi Minh City, Ho Chi Minh City, 700000 Vietnam; 3 My Thien Odonto-Stomatology Hospital, Ho Chi Minh City, 700000 Vietnam

**Keywords:** β-tricalcium phosphate, Platelet-rich fibrin, Alveolar bone graft, Cone beam computed tomography, Residual calcified material

## Abstract

**Background:**

This study aimed to evaluate the effectiveness of β-tricalcium phosphate (β-TCP) and platelet-rich fibrin (PRF) in unilateral alveolar bone graft, involving the percentage of residual calcified material and the average labiopalatal thickness of the grafts on cone beam computed tomography at 6 months after surgery, comparing two age groups 12 years and under and over 12 years old.

**Results:**

The mean preoperative defect volume was 0.93 ± 0.20 cm^3^, with no significant difference between the two groups (*p* = 0.652). In the postoperative period, we did not record any abnormal bleeding and no infection was observed. Six months after surgery, the mean percentage of residual calcified material was 63.53 ± 16.48% with a significantly higher difference in the age group 12 and under (*p* < 0.001), and the mean average labiopalatal thickness of the grafted bone was 5.72 ± 1.09 mm with a significantly higher difference in the age group 12 and under (*p* = 0.011).

**Conclusion:**

Using β-TCP and PRF in alveolar bone graft surgery has acceptable effectiveness clinically and on CBCT images, with significantly higher differences of the percentage of residual calcified material and the average labiopalatal thickness of the grafted bone in the group 12 years old and younger than in the older group.

## Background

Clefts lip and palate are the most common major congenital craniofacial abnomality [1], with an incidence of 1:700 births, of which 75% of individuals present with alveolar cleft [2]. In essence, alveolar cleft is a congenital maxillary defect localized in the anterior tooth region. Untreated alveolar clefts often lead to oronasal fistulae, affecting pronunciation, causing hypoplasia of the maxillary bone, crowding of teeth, and facial asymmetry [3]. Since the 1970s, the first studies on alveolar graft surgery have been reported in the literature and fought becoming a popular method for the treatment of alveolar cleft [3]. Up to now, the autologous iliac crest is still the gold standard graft material in alveolar bone graft surgery [4, 5]. However, the use of autologous bone still has some limitations such as the ability to limit bone volume, difficulty to control the rate of bone resorption. Besides, the patient has to endure an additional surgical area that is painful and invasive, increasing the rate of complications and the cost of treatment for patients [6].

β-tricalcium phosphate (β-TCP) is a bio-ceramic material with the osteoinductive and osteoconductive ability not only does not interfere with physiological teething but also is completely converted into autologous bone after 6 to 24 months. It is a safe, economical and promising solution in alveolar bone graft surgery [6–9]. In addition, the healing efficiency was improved and the rate of bone regeneration was increased when β-TCP was combined with platelet-rich fibrin (PRF) [10, 11]. However, so far, to the knowledge of our research team, there have been no reports on the application of β-TCP and PRF as a graft material in alveolar bone graft. Therefore, this study was conducted to evaluate the effectiveness of β-TCP and PRF in unilateral alveolar bone graft, involving the percentage of residual calcified material and the average labiopalatal thickness of the grafts at 6 months after surgery, comparing two age groups 12 years and under and over 12 years old.

## Methods

### Patients

Twenty-four consecutive patients with unilateral alveolar clefts received alveolar bone graft surgery using β-TCP and PRF at My Thien Odonto-Stomatology Hospital from November 2021 to September 2022. All patients were 8 years or older, were divided into two groups over 12 years old and 12 years old or younger, had primary cheiloplasty, palatoplasty and orthodontic treatment before surgery. Patients with systemic syndromes, craniofacial syndromes, bilateral clefts and previous alveolar grafts were excluded from the study. All patients had informed consent from their parents/guardians to participate this study.

This study was approved by the Ethics Committee in Biomedical Research of University of Medicine and Pharmacy at Ho Chi Minh City on November 19, 2021, No. 619/HDDD-DHYD.

### Design

Prospective, descriptive clinical study.

### Materials

- One hundred percent pure β-TCP (CeraSorb®M Ancladen, Barcelona, Spain) with 65% porosity and 500–1000 μm particle size.
- Membrane platelet-rich fibrin (A-PRF) and liquid (S-PRF) were prepared according to the manufacturer’s procedure (Process for PRF, Nice, France).

### Bone graft preparation

**Fig. 1:**
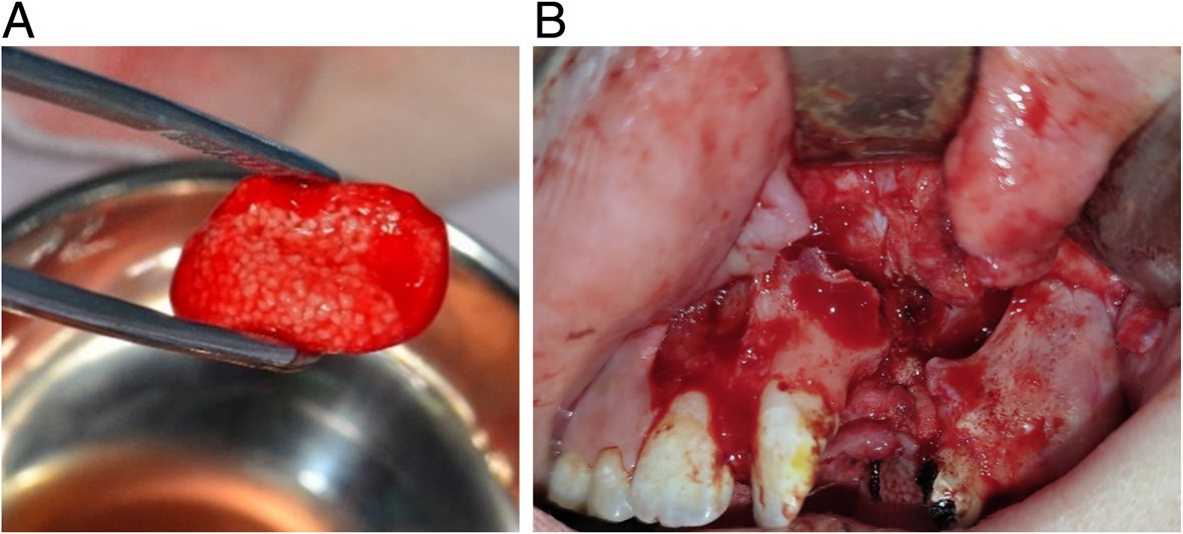
**A** Sticky bone graft. **B** The base of the nose and palate before grafting

The patient’s venous blood was taken into 2 tubes of A-PRF and 2 tubes of S-PRF (about 10 ml of blood/1 tube) rotated at 1300 rpm for 14 min [12]. When the time was up, the blood tubes separated into the red and yellow components. The red part containing mainly red blood cells was discarded. The liquid yellow aliquot of the A-PRF and S-PRF tubes was used to mix with the β-TCP to form sticky bone graft (as shown in Fig. 1A). The yellow solid part of each A-PRF tube was pressed with a film press under suitable pressure to form a PRF membrane of uniform thickness.

### Surgical progress

All of the patients was performed by a surgeon with over 20 years of clinical experience.

The patient was generally anesthetized through an oral endotracheal tube, in the supine position, with the head upright.

The process of flap design and preparation of the transplant recipient area were performed according to the procedure of Craven et al. [13] (as shown in Fig. 1B). The sticky bone graft would be packed into the defect in the following order: a PRF membrane was lined on the base of the nose and palate, the sticky bone graft was compressed into the defect after that and a PRF membrane is lined outside the sticky bone graft [14]. The volume of sticky bone graft used was 30% more than the preoperative defect volume measured on cone beam computed tomography (CBCT) data [15], and we used a 1-cc syringe to measure the amount of material needed. Finally, the flap was relieved by a periosteal incision and closed with Vicryl 4-0 sutures.

The patient was discharged the same day and took antibiotics, anti-inflammatory drugs, and analgesics for 5 days after surgery.

### Data collection

Obtained medical record information of patients after obtaining written consent from the patient’s parent/guardian for participating this research and publication.

**Fig. 2:**
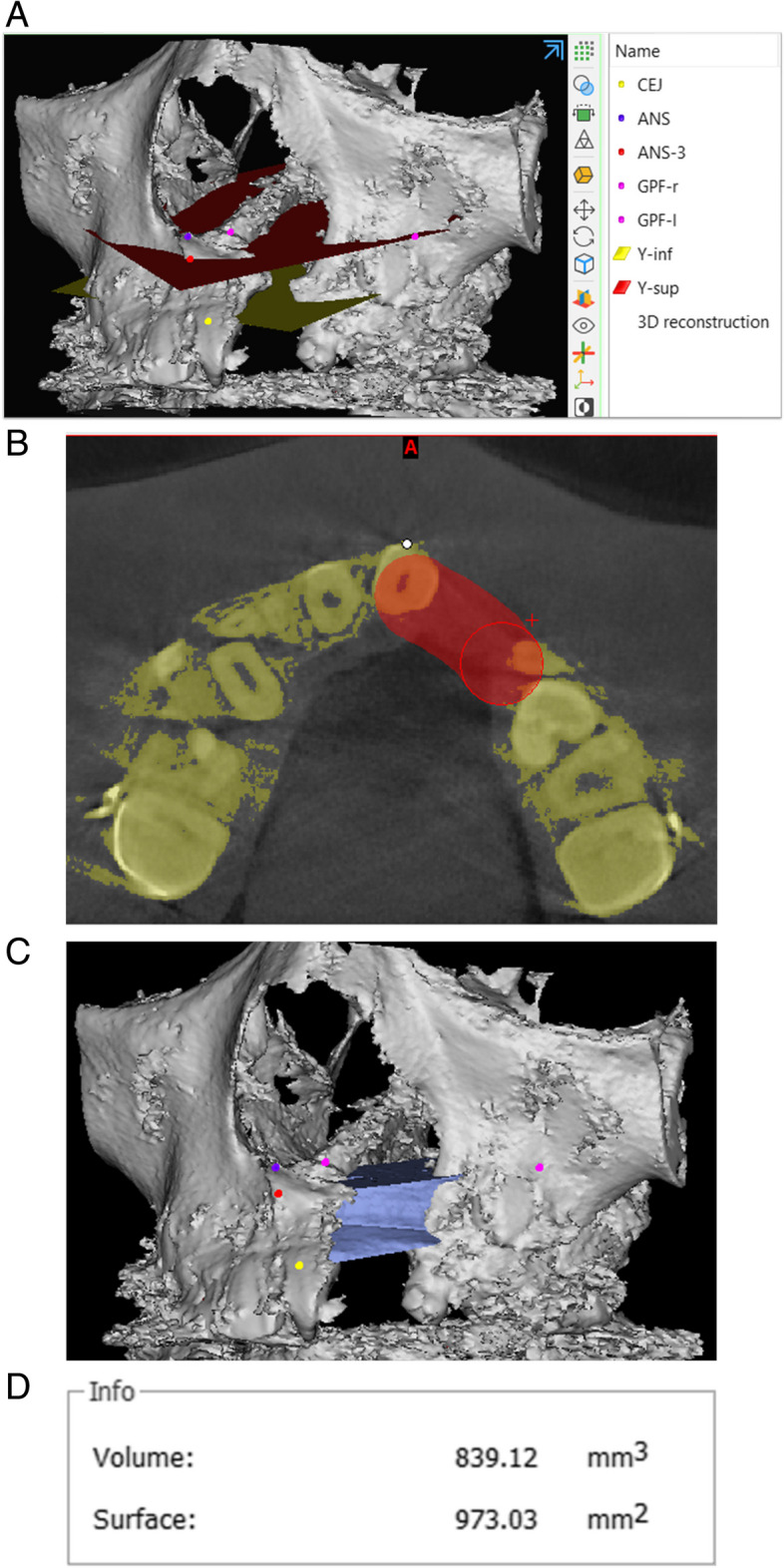
**A** Determination of landmarks and boundary planes. **B** Drawing of the outer (labial) and inner (palatal) boundaries. **C** Simulation of an ideal alveolar cleft
- Stage 2: The gray scale (pseudo Hounsfield Units) of the bone and teeth surrounding the cleft were determined to create the mesial and distal boundaries of the alveolar clefts. This step was done by the commands “Profile line”, “Threshold” in the “Segmentation” section. At the end of this stage, the result would be a simulation of the three-dimensional shape and slices of the bony area surrounding the cleft.
- Stage 3: The inner (palatal) and outer (labial) boundaries of the alveolar clefts are marked in horizontal slices based on the bony margin of the alveolar cleft. Specifically, choose the command “Multiple Slice Edit”, “Auto-interpolate”, select the canvas as “Ellipse” with the size of 8 mm × 8 mm (as shown in Fig. 2B). Then, the outer (labial) boundaríes are drawn by hand once every millimeter according to the mesial and distal osseous margins. The inner (palatal) boundaries shall be defined as parallel and 8 mm from the outer boundaries.
- Stage 4: Finally, the spatial boundaries established from the above stages are combined and created a simulation of the defect using the commands “Cut with plane” and “Boolean operation” (as shown in Fig. 2C). The software would automatically calculate and create a simulation block with a uniform labiopalatal dimension of 8 mm from top to bottom according to the outer (labial) bone margin bounded by the [Y-inf] and [Y-sup] planes. The defect volume is determined by the command “Properties”.

All patients underwent CBCT (Orthophos SL 3D–Densply Sirona) with FOV 11 cm × 10 cm, imaging time 13 s, 60–90 kV and 3–16 mA before surgery and 6 months after surgery. The diagnostic images were reconstructed into DICOM format file sequences. This series of files was analyzed with Mimics 21.0 software to evaluate the preoperative defect volume (V_0_) with the stages described as follows:

- Stage 1: The reference landmarks and planes were marked according to Linderup et al. [16] (as shown in Table 1) with the commands “Point”, “Plan” in the “Analyze” section. The goal of this stage was to determine the upper and lower boundaries of the alveolar clefts (as shown in Fig. 2A).

**Table 1:** The reference landmarks and planes

	Definition
ANS	Point of Anterior nasal spine
ANS-3	Point is 3 mm below ANS
CEJ	The lowest point of the cemento-enamel junction on the labial surface of the cleft side central incisor
GPF-r	Right side greater palatine foramen
GPF-l	Left side greater palatine foramen
[Y-inf]	Plane passing through CEJ, GPF-r, and GPF-l
[Y-sup]	Plane parallel to [Y-inf] and passing through ANS-3

**Fig. 3:**
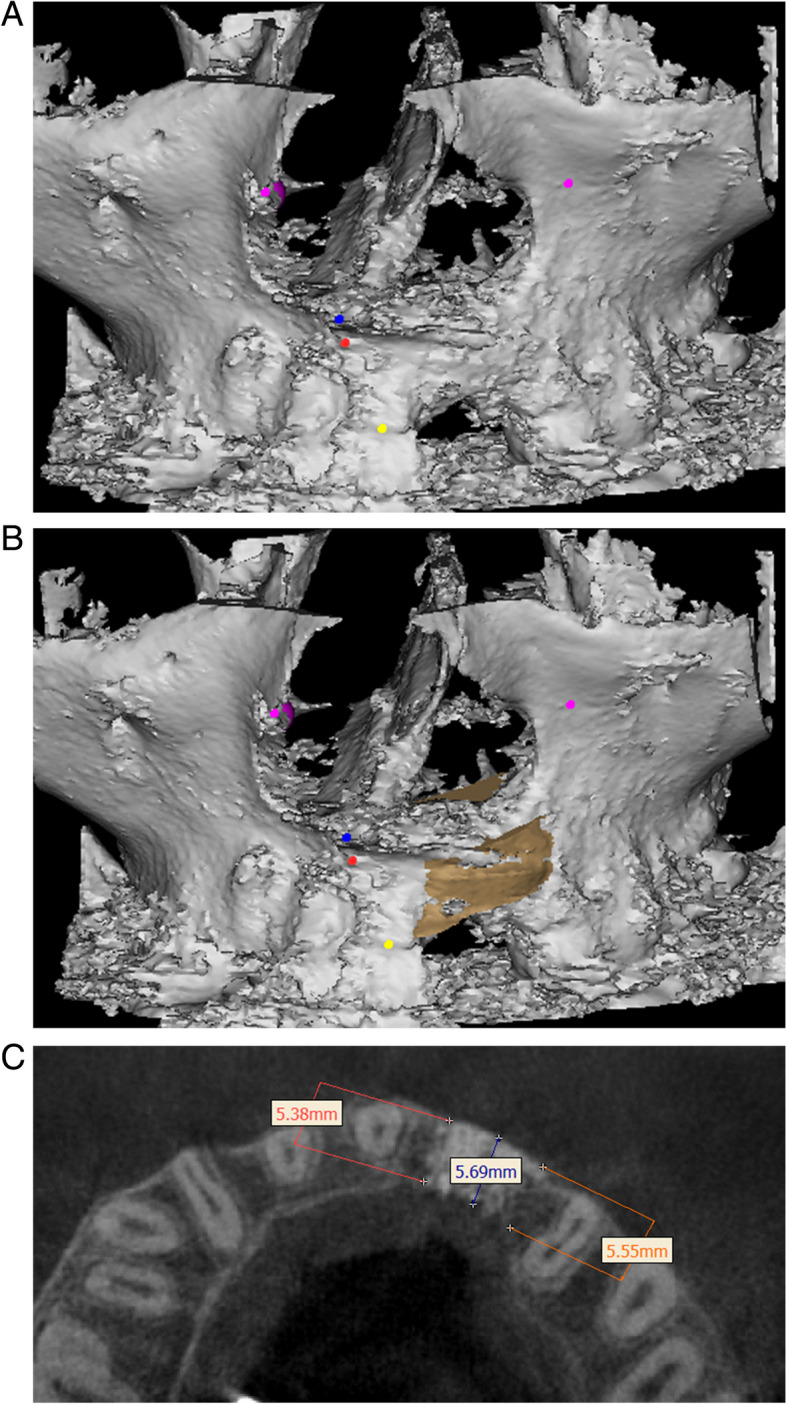
**A** Postoperative 3-D reconstructed CBCT image. **B** Simulation of ideal alveolar cleft. **C** The labiopalatal thickness of the graft

Measure the defect volume at 6 months postoperative (V_1_) using the same method as preoperative measurements (as shown in Fig. 3A, B). The percentage of residual calcified material is the result of the calculation: (V_0−_V_1_)/V_0_

The average labiopalatal thickness of the graft in the horizontal plane was evaluated according to the study of Suomalainen et al. [17] (as shown in Fig. 3C), with the stages described as follows:

- Stage 1: Determine the Frankfort plane by the external auditory point on both sides and the infraorbital point on the cleft side. Set up horizontal slides parallel to the Frankfort plane.
- Stage 2: Identify the parts of the root of the permanent central incisor on the cleft side, define three points:
  - Point of root neck: about 1 mm from the highest point of the outer cementum-enamel junction to the apex.
  - Point of root apex: corresponds to the root apex.
  - Midpoint of tooth root: midpoint of two points mentioned above.
- Stage 3: On the horizontal slides passing through these points, measure the labiopalatal thickness at the mesial, middle and distal positions of the cleft. In the end we have 9 values, and the average labiopalatal thickness of the graft is the average of those values.

In our study, measurements were performed by an independent evaluator with an intraclass correlation coefficient of 0.94.

### Statistical analysis

Variables were statistically analyzed and compared using independent sample *t*-test, *p* < 0.05 was considered statictical significance.

## Results

A total of 24 patients with unilateral alveolar clefts participated in this study. The age of the patients varied from 7 to 18 years, with the mean age being 13.5 ± 4.8 years, of which 45.8% (11/24) of the patients were over 12 years old. In terms of gender, the percentage of women was higher than that of men (54.2% versus 45.8%). The mean preoperative defect volume was 0.93 ± 0.20 cm^3^, with no significant difference between the two groups (*p* = 0.652) (as shown in Table 2).

**Table 2:** Results on CBCT data analysis

Variables	Mean ± SD			p*
	≤ 12 years old (*n* = 13)	> 12 years old (*n* = 11)	Total	
Preoperative defect volume (cm^3^)	0.93 ± 0.21	0.93 ± 0.20	0.93 ± 0.20	0.652
Percentage of residual calcified material 6 months postoperative (%)	73.98 ± 8.77	51.18 ± 14.92	63.53 ± 16.48	< 0.001
Average labiopalatal thickness of the graft 6 months postoperative (mm)	7.28 ± 0.98	4.17 ± 0.91	5.72 ± 1.09	0.011

*Independent sample *t*-test

In the postoperative period, we did not record any abnormal bleeding and no infection was observed, except for two cases (one in each group) where a few amount of particulate material was dropped through the nose. Analysis of CBCT data showed that 6 months after surgery, the mean percentage of residual calcified material was 63.53 ± 16.48% with a significantly higher difference in the age group 12 and under (73.98 ± 8.77%) than in the age group over 12 (51.18 ± 14.92%) (*p* < 0.001), and the mean average labiopalatal thickness of the grafted bone was 5.72 ± 1.09 mm with a significantly higher difference in the age group 12 and under (7.28 ± 0.98 mm) than in the age group over 12 (4.17 ± 0.91 mm) (*p* = 0.011) (as shown in Table 2).

## Discussion

In our study, two children (one in each group) were noted to have small amounts of particulate leakage after surgery, all of which were in the nasal passages. One case over 12 years old appeared 7 days after surgery, one case under 12 years old appeared about 2 months after surgery. These two cases were reported by the patients and parents, and upon re-examination, they were not detected. Janssen et al. also noted this phenomenon in some cases but did not affect the final result [6]. However, alveolar bone graft surgery is a complex technique, performed by many authors in different ways. In this study, we created a flap as described by Craven et al. to maximize the coverage of the periosteum and keratinized gingiva on the graft [13]. Another challenge encountered during alveolar bone graft surgery is that suturing the nasal base flap and the palatal flap was difficult to perform. The reason is because the surgical field is very narrow, the mucosa of the nasal base is thin, the shape of the bone base is very variable in each child, and the high technical standards for each stitch, especially the apex position of the nasal base and palatal flap [18]. Therefore, we placed an additional layer of A-PRF membrane lining the nasal and palatal base to increase the coverage of the graft.

The alveolar bone graft surgery procedure in this study required 4 tubes of blood, equivalent to about 40 ml of blood. According to Balaji, the average amount of blood lost of surgical sites of alveolar cleft is about 60 ml [19]. Thus, the total blood loss is estimated at about 100 ml. When comparing with a conventional alveolar bone graft—autologous bone grafting from the iliac crest—the average total blood loss was 132 ml [19]. Therefore, it represented that alveolar bone grafting with β-TCP and PRF is less blood loss and less invasive beacause there is no donor site of the iliac crest.

The literature noted that variability in the percentage of residual calcified material at 6 months after surgery is significant between studies. This difference is attributed to the lack of uniformity in study design methods (age, graft material) and measurement methods (slice thickness, reference anatomical landmarks, softwares, and measuring methods). A systematic review and meta-analysis reported an average of 63.38% residual calcified tisue at 1 year after alveolar bone grafting with cancellous iliac crest [20]. The study using β-TCP reported that the percentage of residual calcified material at 6 months and 1 year after surgery were 73 ± 6% and 65 ± 14%, respectively [4, 6]. In our study, the average percentage of residual calcified material at 6 months after surgery was 63.53%; but when assessing the group of children under 12 years old, this rate was up to 73.98%. This illustrated that the timing of alveolar bone grafting is very important. Some authors hypothesized that the pressure created by the growing teeth into the graft site is necessary to ensure the physiological function of the graft, thereby helping to limit the loss of the residual calcified material [13]. On the other hand, the growth of periodontal tissue around the growing teeth at the graft site also contributes to the increase of the graft volume [21].

One of the factors to consider when evaluating alveolar bone grafting is the average labiopalatal thickness of the graft. Stoop et al. reported that the labiopalatal thickness should be at least 8 mm to ensure the eruption of canines into the bone grafting area [22]. This viewpoint derives from reports of adult alveolar bone size at the canine position and the maximum labiopalatal dimension of canines, usually 8 mm [23, 24]. Therefore, we chose 8 mm as the ideal labiopalatal thickness to determine the preoperative defect volume. However, applying this value as a criterion for alveolar bone grafting surgery in children is not really satisfactory because the bone size of children is always smaller than that of adults. On the other hand, the movement of teeth into the alveolar region creates pressure that can increase the labiopalatal dimension up to 1.5 mm [25]. In addition, some authors reported that the labiopalatal thickness of the graft required to ensure success for orthodontic treatment is at least 5 mm [26]. Our study recorded this index reaching 5.72 ± 1.09 mm at 6 months after surgery. This is similar to the studies using cancellous bone and granular grafts of Seike et al. [26] and Alchalabi et al. [27]. This result showed that the combination of β-TCP and PRF has a good effect in alveolar bone graft surgery.

## Conclusion

Using β-TCP and PRF in alveolar bone graft surgery minimizes invasiveness for patients. This technique has acceptable effectiveness clinically and on CBCT images, with significantly higher differences of the percentage of residual calcified material and the average labiopalatal thickness of the grafted bone in the group 12 years old and younger than in the older group.

## Data Availability

Data and material are available on request.

## Abbreviations

β-TCP β: Tricalcium phosphate
PRF: Platelet-rich fibrin
CBCT: Cone beam computed tomography
DICOM: Digital Imaging and Communications in Medicine
